# Modeling hormonal and inflammatory contributions to preterm and term labor using uterine temporal transcriptomics

**DOI:** 10.1186/s12916-016-0632-4

**Published:** 2016-06-13

**Authors:** Roberta Migale, David A. MacIntyre, Stefano Cacciatore, Yun S. Lee, Henrik Hagberg, Bronwen R. Herbert, Mark R. Johnson, Donald Peebles, Simon N. Waddington, Phillip R. Bennett

**Affiliations:** Imperial College Parturition Research Group, Institute of Reproduction and Developmental Biology, Imperial College London, Hammersmith Campus, London, United Kingdom; Perinatal Center, Department of Clinical Sciences, University of Gothenburg, Gothenburg, Sweden; Centre for the Developing Brain, Division of Imaging Sciences and Biomedical Engineering, King’s College London, King’s Health Partners, St. Thomas’ Hospital, London, United Kingdom; Academic Department of Obstetrics and Gynaecology, Chelsea and Westminster Hospital, London, United Kingdom; UCL Centre for Perinatal Brain Protection & Repair, Institute for Women’s Health, University College London, London, United Kingdom; Gene Transfer Technology Group, Institute for Women’s Health, University College London, London, United Kingdom; Antiviral Gene Therapy Research Unit, Faculty of Health Sciences, University of the Witswatersrand, Johannesburg, South Africa

**Keywords:** Preterm labor, Inflammation, Progesterone, Myometrium, Transcriptome, RNA-Seq

## Abstract

**Background:**

Preterm birth is now recognized as the primary cause of infant mortality worldwide. Interplay between hormonal and inflammatory signaling in the uterus modulates the onset of contractions; however, the relative contribution of each remains unclear. In this study we aimed to characterize temporal transcriptome changes in the uterus preceding term labor and preterm labor (PTL) induced by progesterone withdrawal or inflammation in the mouse and compare these findings with human data.

**Methods:**

Myometrium was collected at multiple time points during gestation and labor from three murine models of parturition: (1) term gestation; (2) PTL induced by RU486; and (3) PTL induced by lipopolysaccharide (LPS). RNA was extracted and cDNA libraries were prepared and sequenced using the Illumina HiSeq 2000 system. Resulting RNA-Seq data were analyzed using multivariate modeling approaches as well as pathway and causal network analyses and compared against human myometrial transcriptome data.

**Results:**

We identified a core set of temporal myometrial gene changes associated with term labor and PTL in the mouse induced by either inflammation or progesterone withdrawal. Progesterone withdrawal initiated labor without inflammatory gene activation, yet LPS activation of uterine inflammation was sufficient to override the repressive effects of progesterone and induce a laboring phenotype. Comparison of human and mouse uterine transcriptomic datasets revealed that human labor more closely resembles inflammation-induced PTL in the mouse.

**Conclusions:**

Labor in the mouse can be achieved through inflammatory gene activation yet these changes are not a requisite for labor itself. Human labor more closely resembles LPS-induced PTL in the mouse, supporting an essential role for inflammatory mediators in human “functional progesterone withdrawal.” This improved understanding of inflammatory and progesterone influence on the uterine transcriptome has important implications for the development of PTL prevention strategies.

**Electronic supplementary material:**

The online version of this article (doi:10.1186/s12916-016-0632-4) contains supplementary material, which is available to authorized users.

## Background

Preterm labor (PTL), defined as labor onset before 37 weeks of gestation, is the leading cause of neonatal mortality in children under 5 years of age, with incidence rates continuing to rise in both developing and developed countries [1]. PTL is a complex syndrome resulting from the activation of multiple pathological processes; however, infection and inflammation are implicated in the majority of cases of very early PTL in humans, whereas they are not in most late PTL cases [2, 3]. Regardless of the causal factor(s) determining the timing of labor onset, both preterm and term parturition involves common phenotypic changes, including cervical remodeling and dilatation, fetal membrane rupture, and uterine contractions that collectively permit the expulsion of the fetus [4].

The transition of the uterus from a quiescent to contractile organ results from a complex interplay between inflammatory and hormonal signals yet the relative contribution of each to the contractile phenotype remains poorly understood. In the mouse, parturition at term appears to be initiated by fetal lung maturation, which drives steroid receptor cofactor-dependent secretion of surfactant proteins into the amniotic fluid, migration of activated macrophages into the uterus, and induction of the inflammatory transcription factor nuclear factor kappa light chain enhancer of activated B cells (NFkB). This leads to the upregulation of inflammatory and contraction-associated genes within the uterus, including those encoding for the principal prostaglandin synthase enzyme, cyclooxygenase-2 (COX-2). Increased levels of prostaglandin F2 alpha (PGF_2α_) cause luteolysis and subsequent progesterone withdrawal, leading to further activation of pro-labor pathways [5–8]. However, stimulation of uterine contractions and preterm birth can be achieved in the mouse in the absence of progesterone withdrawal using bacteria or bacteria-derived products (e.g., lipopolysaccharide, LPS) [9, 10]. We have also recently shown that uterine activation of the inflammatory transcription factor activator protein 1 (AP-1), and not NFkB, is sufficient to stimulate inflammatory and contractile gene transcription and pathways causing PTL in the mouse at gestation day 16, when circulating progesterone levels are high [11–13]. While these findings indicate that pathophysiological PTL and spontaneous term labor in the mouse involve modulation of different hormonal and inflammatory pathways, their convergence in a common contractile phenotype suggests overlap and redundancy in the underlying mechanisms of parturition.

As in the mouse, human labor is accompanied by infiltration of immune cells into gestational tissues and release of inflammatory mediators that promote cervical ripening and myometrial activation [14]. Unlike most other mammals, this occurs in the presence of high levels of circulating progesterone, suggesting that inflammatory pathway activation in the human uterus may play a dominant role in promoting the laboring phenotype [15–17]. However, although progesterone concentrations in women remain high beyond labor, a number of non-mutually exclusive hypotheses have been formulated to explain how a “functional” withdrawal of progesterone may still precede inflammatory activation at term and signal parturition onset in humans: (1) catabolism of progesterone in the uterus into metabolites that regulate relaxant pathways independent of progesterone receptor (PR) action [18]; (2) altered expression of PR isoforms that drive upregulation of inflammatory mediator expression [19–21]; (3) changes in cofactor protein levels affecting PR transactivation [22]; and (4) inflammation-induced trans-repression of PR by NFkB [23]. In addition, recent mouse model investigations of immune cells in gestational tissues suggest that parturition-associated inflammation may not be causative of labor, but may be involved in the amelioration of host response to pathogens and the activation of tissue repair mechanisms necessary for remodeling of the uterus following delivery [24–28].

Many of the hormonal and inflammatory pathways proposed to regulate term and preterm human parturition can be recapitulated in the mouse. Inhibition of the PR by the antagonist pharmaceutical drug RU486 (mifepristone) robustly induces PTL in the mouse and the same molecule acts as an effective abortifacient in humans [29, 30]. Infection and/or inflammation are frequently associated with human PTL, accounting for 30 % of all cases [2, 31]. Activation of cytokine and inflammatory pathways associated with human PTL are similarly stimulated in infection/inflammation-induced PTL mouse models [11, 12, 32–34]. The use of these mouse models also permits temporal characterization of parturition-associated gene expression changes in the uterus, which are manifested prior to the acquisition of the contractile phenotype. Previous time-course studies of gene expression changes in the murine pregnant uterus using microarray have been highly informative yet limited by the comparatively low specificity, sensitivity, and resolution of hybridization-based techniques [35–37]. Furthermore, practical and ethical reasons limit the feasibility of conducting similar studies in the human and thus studies on gestational tissues (myometrium, cervix, and chorioamniotic membranes) have been limited to the comparison of samples collected before and after labor onset [38–44]. An absence of time-course data from human studies has further limited our knowledge of large-scale gene expression changes involved in initiating the onset of labor, most of which are likely to be manifested some time prior to labor.

In this study we use RNA sequencing (RNA-Seq) to temporally characterize global uterine transcriptome changes in three models of mouse parturition, (1) spontaneous term labor, (2) inflammation-induced PTL, and (3) RU486-induced progesterone withdrawal leading to PTL, to identify transcriptomic changes specific to each model and the core set of genes essential to parturition in all circumstances. We assess the relative contribution of progesterone-regulated and inflammatory-regulated transcription pathways to the mechanism of labor in each of the murine models and compare these changes to human uterine transcriptome data.

## Methods

### Murine studies and ethics statement

All animal studies were performed under UK Home Office License 70/6906 in accordance with the UK Animals Scientific Procedures Act of 1986 and with approval of the Imperial College and University College London Ethical Review Committees. Virgin female CD1 outbred mice were time mated and the presence of a copulatory plug was considered day 0 of gestation. Mice were housed in open cages at 21 ± 1 °C under a constant 12 h light-dark cycle regimen, with ad libitum access to standard rodent food and water. Unless otherwise stated, each experiment represents a minimum of four biological replicates.

### Murine models of preterm labor

For the infection/inflammation-induced PTL murine model, CD1 pregnant mice received, at gestational day 16 (E16), a subcutaneous injection of morphine (2.5 mg/kg) 20 min before the surgery. A laparotomy was performed under general anesthesia using isoflurane as previously described [11, 12]. Uterine horns were exteriorized and 20 μg (25 μL total volume in phosphate-buffered saline [PBS]) of *Escherichia coli* LPS serotype O111:B4 (Sigma) or sterile PBS was injected into the upper right uterine horn between the first and the second sacs without entering the amniotic cavity. Recovery following surgery was remotely monitored via an infrared CCTV camera system until tissue collection or the onset of spontaneous delivery. Onset of labor was defined as delivery of the first pup. For the noninflammatory model of PTL induced by chemical progesterone withdrawal, CD1 pregnant mice received, at E16, a 150 μg subcutaneous injection of RU486 (Sigma) or vehicle (20 μl dimethyl sulfoxide [DMSO]) as described previously [11, 45]. Animals were monitored by infrared CCTV prior to tissue collection or the onset of labor.

### Tissue collection

Briefly, tissue samples from the upper right uterine horn were excised and immediately snap-frozen on dry ice before being stored at −80 °C until processing for RNA extraction. For the gestational model, samples were harvested at E14, E16, E18, and during active term labor (LAB). For the RU486-induced PTL model, tissue was collected 6 h and 18 h post injection (hpi) or during active labor (20 ± 1.6 hpi, mean ± SD), corresponding respectively to 30 %, 90 %, and 100 % of the time between the injection and the onset of PTL. Similarly for the LPS-induced PTL model, myometrium was collected 2 h and 6 h following PBS or LPS injection and during active labor (7 ± 2 hpi, mean ± SD), which again was designed to correspond to 30 %, 90 %, and 100 % of the time interval between injection and PTL onset.

### RNA isolation and sequencing

Frozen myometrium tissue was pulverized in liquid nitrogen and RNA was extracted in lysis buffer ML (guanidinium thiocyanate) using the NucleoSpin® miRNA kit (Macherey-Nagel) with DNase treatment following manufacturer’s instructions. RNA quality was assessed for all samples using an RNA 6000 Nano Kit and Bioanalyzer (Agilent). Samples with an RNA integrity number (RIN) >8 were used for RNA-Seq library preparation. cDNA libraries with an insert length of 300 bp were prepared from 2 μg of purified large RNA fraction (>200 nucleotides) using a TruSeq Stranded mRNA Sample preparation kit (Illumina). Samples were enriched for poly-A mRNA using oligo-dT-coated magnetic beads. Following purification, mRNA was randomly fragmented at 94 °C for 8 min to obtain fragments of around 200 bp while minimizing bias at the 3' end of transcripts. First-strand complementary DNA synthesis was performed using random primers (Illumina) and SuperScript II Reverse-Transcriptase (Invitrogen) followed by second strand synthesis with RNaseH and DNA polymerase I (Illumina). Adapters provided were used to tag each sample. Compatibility between adapters was checked with Illumina Experiment Manager to allow subsequent pooling of three samples in each lane during sequencing. cDNA libraries were amplified by 10 cycles of PCR. The quality of each library was evaluated on a 2100 Bioanalyzer (Agilent) followed by paired-end sequencing (2 × 100 bp) on an Illumina HiSeq 2000. Sample preparation and run was randomized by model and treatment.

### Processing of RNA-Seq data

RNA-Seq reads were aligned to the reference genome of *Mus musculus* GRcm38 from ENSEMBL release 73 using the software package Burrows-Wheeler Aligner (v0.75a) [46]. The command line utility Picard was used to sort the resulting BAM alignment files [47]. Samples were quality assessed using FastQC [48]. Gene level read counts were generated in R using the GenomicRanges and Rsamtools Bioconductor packages against the *Mus musculus* GTF annotation file version GRCm38 (release 73)[49–51]. A total of 269 gigabytes of sequencing data from 57 samples were generated with an average 87.9 million reads per myometrium sample (Additional file 1). Outliers were detected based on the distance from the centroid: for more information and the R script see Additional file 2. While no outliers were detected in the term gestation model, two samples were classified as outliers (*P* < 0.01, Bonferroni post hoc test) in the RU486- and LPS-induced PTL models (6 h DMSO and 6 h LPS) and excluded from further analysis.

### Differential expression analysis of gene expression data

Gene read counts were normalized for the library size using TMM scaling implemented in the Bioconductor package followed by transformation into log_2_ counts per million. Differentially expressed genes (DEGs) were identified in R using the negative binomial differential expression method edgeR [52]. *P* values were adjusted for multiple testing with the Benjamini–Hochberg correction and a false discovery rate (FDR) cutoff of 0.001 was used. The full gene expression dataset is reported in Additional file 3.

### Pathway analysis

Biological functions enriched with DEGs were identified using two manually curated databases of signaling and metabolic pathways represented on networks (Process Network Analysis by MetaCore®, Thomson Reuters [53]) or on canonical pathways maps (canonical pathways analysis by Ingenuity® Pathway Analysis [IPA®], QIAGEN Redwood City).

Process Network Analysis was performed to identify biological functions significantly enriched by DEGs. A threshold *P* value less than 0.05 (−log_10_ = 1.3) was used as a cut-off. Overrepresented canonical pathways were also identified using IPA (QIAGEN Redwood City), which, in addition to identifying enriched biological functions, allows prediction of the activation status for each specific pathway. Canonical pathways were considered significantly enriched when *P* < 0.05 and activated/inhibited when Z-score ≥ |2.0|.

### Causal Network Analysis

IPA was used to identify upstream regulators potentially responsible for the observed gene expression changes. Putative upstream regulators were predicted using IPA Causal Network Analysis by calculating a regulation Z-score and an overlap *P* value, which predict the activation or inhibition state of regulators based on downstream DEGs and known directionality of expression [54]. Upstream regulators with overlap *P* ≤ 0.05 and an IPA activation Z-score ≥ |2.0| were considered significantly activated/inhibited. The overlap *P* value (calculated used Fisher’s exact test) threshold was used to identify possible upstream regulators based on significant overlap between DEGs and known targets regulated by a given upstream regulator. The activation Z‐score was used to infer likelihood of activation status of upstream regulators based on evidence from DEGs in our dataset for which the direction of the observed expression change was consistent with that expected from the literature.

### Multivariate data analysis

For multivariate analysis, genes with zero read counts were excluded and the remaining genes were mean-centered and unit variance-scaled. Principal component analysis (PCA) was performed on transcriptome changes using the standard algorithm as implemented in the prcomp function included in the R library *stats*. Transcriptome changes specifically associated with progression of gestation characterizing each murine model were identified and modeled using orthogonal signal correction-partial least squares (OSC-PLS) regression modeling [55]. OSC-PLS is a supervised method that aims to maximize the variance between groups in the output data (i.e., score). It also provides as output an indication (i.e., loadings) of the importance of the variables in the regression. Analyses were carried out in R and OSC-PLS modeling was applied to expression levels of 4142 genes (FDR < 0.001), including those changing in at least one pairwise comparison within a model. Loadings values indicating the relative importance of each gene to each murine model of gestation are reported in Additional file 4. Model goodness of fit was assessed by calculating the correlation coefficients (*r*) using Pearson linear correlation and *P* values were also calculated for each prediction. Hierarchical clustering was performed using Ward’s method [56]. The number of clusters was calculated using the Silhouettes algorithm [57].

### Analysis of human myometrium RNA-Seq data

A human myometrium RNA-Seq dataset deposited by Chan et al. [40] was re-analyzed in our study to permit comparison of our murine transcriptome data with relevant human data. This dataset was derived from samples of lower segment human myometrium collected during Caesarean section at term (38–40 weeks of gestation) from women in active, spontaneous labor (*n* = 5, IL) and from women not in labor undergoing elective Caesarean section (*n* = 5, NIL). Women in the IL group required Caesarean section for undiagnosed breech or cephalopelvic disproportion, without labor augmentation. Count data were accessed at the NCBI Gene Expression Omnibus database using the series accession number [GEO: GSE50599] and genes differentially expressed when comparing IL versus NIL were identified using edgeR with an FDR value <0.001.

Orthologous genes were identified by comparing DEGs associated with human labor onset to murine myometrial transcriptome data in at least one of the following pairwise comparisons: E16 vs E18, E16 vs LAB, and E18 vs LAB for the term gestation model; 6 h RU486 vs 18 h RU486, 6 h RU486 vs LAB_RU486_, and 18 h RU486 vs LAB_RU486_ for the RU486-induced PTL model; 2 h LPS vs 6 h LPS, 2 h LPS vs LAB_LPS_, and 6 h LPS vs LAB_LPS_ for the LPS-induced PTL model. These gene sets were further analyzed using OSC-PLS modeling to test for congruence between human and murine datasets across a time course leading to the acquisition of a laboring phenotype. In the mouse, term labor occurred at gestational day E18.5, corresponding to a human term gestation of approximately 270 days or 38.5 weeks. E18 corresponded to 263 days or 37.5 weeks of human gestation and thus, in the absence of labor, was considered equivalent to the NIL human sample group (term, not in labor). LPS and RU486 treatments led to PTL at E16 within 7 and 20 h following injection, respectively, corresponding to 234 days or 33 weeks of human gestation (preterm). Temporal gene expression changes relative to each murine model of gestation were alternatively used as a training set against which the human datasets were interrogated using OSC-PLS modeling. Pearson correlation scores and *P* values were derived for each test. A positive Pearson score was indicative of a positive correlation between genes similarly changing as a function of gestational age in the mouse and in the human samples. Loadings derived from the OSC-PLS model were indicative of the importance of variables (genes) for discrimination between different sample groups. Hierarchical clustering of loadings was then performed to identify profiles consistent between human and murine gestational models and to identify gene clusters similarly expressed across gestational time course in both organisms.

## Results

### Myometrial transcriptome modeling of term and preterm labor induced by progesterone withdrawal or inflammation

**Fig. 1:**
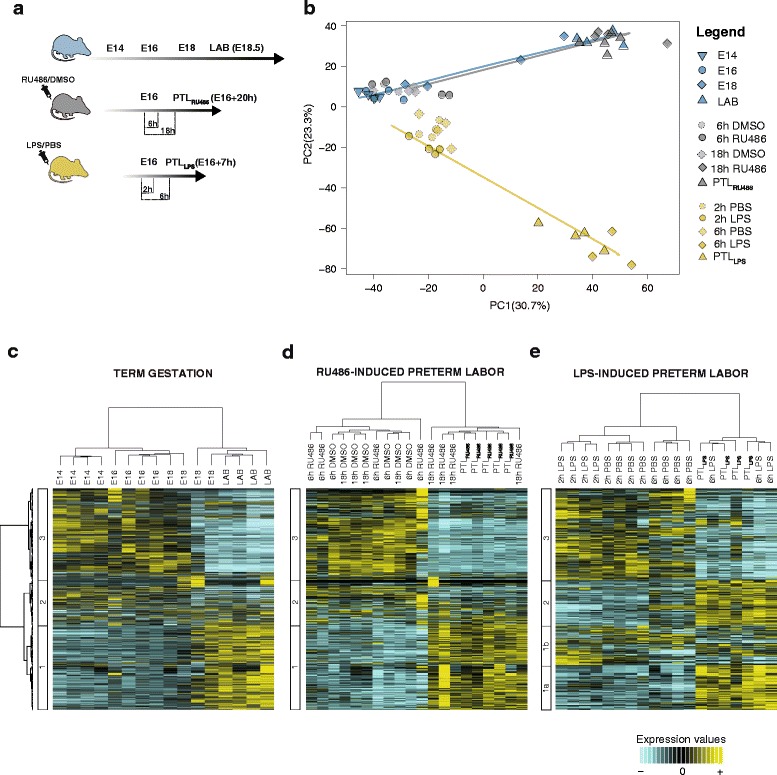
Transcriptome changes associated with term gestation, RU486-, and lipopolysaccharide (*LPS*)-induced preterm labor (*PTL*) in the mouse myometrium. **a** Myometrium samples were collected from three mouse models of gestation: term labor (*blue*), progesterone withdrawal/RU486-induced PTL) (*gray*), and inflammatory/LPS-induced PTL (*yellow*). **b** Principal component analysis of transcriptome changes represented along principal component (*PC*)1 (30.7 % of total variance) and PC2 (23.3 % of total variance). Both term labor and RU486-induced PTL exhibit similar trajectories of transcriptome changes whereas animals experiencing LPS-induced PTL undergo a divergent trajectory. **c**–**e** Heatmaps representing hierarchical clustering analysis performed on differentially expressed genes (false discovery rate < 0.001) for unsupervised identification of gene expression patterns characterizing mouse myometrium collected from term labor (**c**), RU486-induced PTL (**d**), and LPS-induced PTL (**e**). The number of clusters was calculated using the Silhouettes algorithm. (*n* ≥ 4 mice for each experimental group). *DMSO* dimethyl sulfoxide, *LAB* active term labor, *PBS* phosphate-buffered saline

Using RNA-Seq we undertook temporal transcriptome profiling on mouse myometrial samples collected across a time series from animals experiencing spontaneous term labor, or PTL following pharmacologically induced (RU486) progesterone withdrawal, or inflammation-induced (LPS) PTL (Fig. 1a). In normal mouse gestation, circulating progesterone levels peak on E16 before falling to the lowest level on E18 [13]. We therefore characterized the myometrial transcriptome in this model on E14, E16, E18 prior to labor, and E18.5 after the onset of spontaneous labor (postdelivery of the first pup). In the PTL models, LPS and RU486 induced labor onset 7 and 20 hours posttreatment respectively.

Because gene expression changes underpinning the acquisition of the laboring phenotype occur across dramatically different time scales in each of the models, we next assessed transcriptome profiles at time points proportionally comparable on the time course to parturition in each model. Samples were thus collected at 2 and 6 hpi of LPS, and 6 and 18 hpi of RU486. PCA of myometrial transcriptome data revealed that both spontaneous term labor and PTL following progesterone withdrawal involve similar trajectories of transcriptome changes preceding labor. However, a divergent trajectory of myometrial transcriptome changes was observed in animals undergoing inflammation-induced PTL (Fig. 1b). Broad visualization of each individual transcriptome signature indicated consistency within each experimental group; however, in animals delivering at term, there was variation in the myometrial transcriptome just prior to labor on E18, with two samples clustering with E16 and one sample clustering with LAB (Additional file 5). The majority of uterine transcriptome changes were observed to occur between 6 and 18 hpi in the RU486-induced PTL model and between 2 and 6 hpi in the LPS-induced PTL model.

We next performed hierarchical clustering analysis on the 4142 genes differentially expressed (FDR < 0.001) in at least one condition within each of the murine models. Venn diagrams depicting the total number of DEGs for each comparison within each model were also generated (Additional file 6). Comparisons not exhibiting significant gene changes (6 h DMSO vs 6 h RU486 and 6 h LPS vs LAB_LPS_) were excluded from further analyses. A characteristic change in the myometrial transcriptome was detected with increasing gestation and spontaneous term labor onset (Fig. 1c). Three main clusters of gene expression patterns were identified, representing genes either increasing (Cluster 1) or decreasing (Cluster 3) with proximity to labor, or genes exhibiting variable change (Cluster 2). Similar clusters were observed in animals undergoing RU486-induced PTL (Fig. 1d). In contrast, unique transcriptome changes were identified in animals experiencing LPS-induced PTL (Fig. 1e). Cluster 1 could be subdivided into two groups: (1) genes whose expression increased only with the onset of labor (Cluster 1a) and (2) genes whose expression increased immediately after administration of LPS or PBS but decreased following labor onset (Cluster 1b), likely to represent those genes affected by the laparotomy procedure rather than the treatment itself. Consistent with normal term and RU486-induced labor, expression of Cluster 3 genes in the LPS model decreased with approaching labor. Cluster 2 genes were uniquely upregulated in the LPS model just prior to labor onset. Process Network Analysis [58] (MetaCore®, Thomson Reuters) of Cluster 2 genes showed enrichment of pathways associated with cellular adhesion – platelet endothelium–leucocyte interactions (*P* = 8.70 × 10^−9^), proteolysis and connective tissue degradation (*P* = 1.53 × 10^−6^), and enrichment of multiple gene pathways associated with inflammation (immune response – T_H_17-derived cytokines, *P* = 2.25 × 10^−3^; inflammation – IL-6 signaling, *P* = 3.57 × 10^−3^; inflammation – kallikrein–kinin system, *P* = 3.99 × 10^−3^; and immune response – phagocytosis, *P* = 1.02 × 10^−2^) (Additional file 7).

### Identification of a core set of differentially expressed genes associated with term and preterm labor

**Fig. 2:**
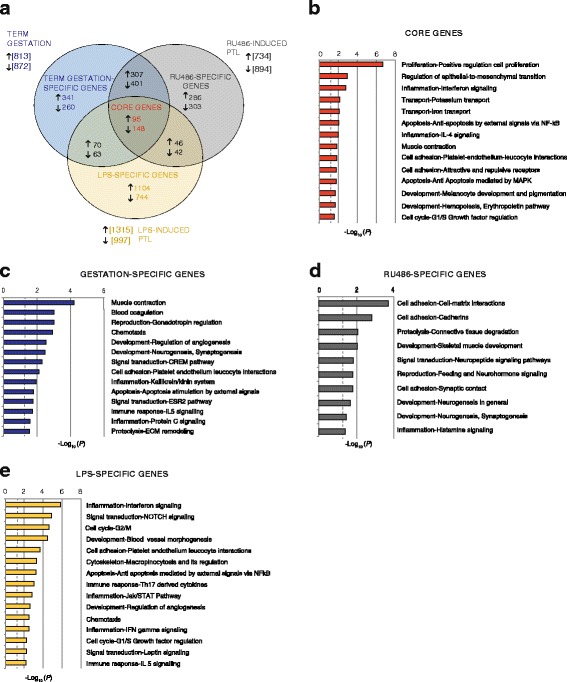
Identification of core gene sets differentially expressed in spontaneous term labor and preterm labor (*PTL*) models. (**a**) Venn diagram representing overlap between differentially expressed genes (*DEGs*) as detected in term labor (*blue*), and RU486-induced (*gray*) and lipopolysaccharide (*LPS*)-induced (*yellow*) PTL models. Process Network Analysis (Metacore®) was performed on the core set, including those genes similarly changing in all models of parturition (**b**), on genes changing only in the term labor model (**c**), genes exclusively changing with RU486 treatment (**d**), and on genes exclusively changing following LPS treatment (**e**). Bar length indicates significance and is equal to the negative logarithm of enrichment *P* value. A *P* value cut-off less than 0.05 (−log_10_ *P* > 1.3) was applied and is indicated by the *dashed line*

To identify sets of genes mutually regulated between models, or unique to each of them, we evaluated the overlap between DEGs detected in the following pairwise comparisons: E16 vs E18, E16 vs LAB, and E18 vs LAB for the term gestation model; 6 h RU486 vs 18 h RU486, 6 h RU486 vs LAB_RU486_, and 18 h RU486 vs LAB_RU486_ for the RU486-induced PTL model; and 2 h LPS vs 6 h LPS, 2 h LPS vs LAB_LPS_, and 6 h LPS vs LAB_LPS_ for the LPS-induced PTL model (Fig. 2a). Canonical pathway analysis of the DEGs was then performed using IPA (QIAGEN Redwood City, www.qiagen.com/ingenuity) to enable identification of groups of biological functions similarly activated or inactivated across these comparisons. A total of 46 canonical pathways were detected as significantly overrepresented in all models of parturition (Additional files 8 and 9). Strong similarity in enrichment profiles characterizing genes changing with spontaneous labor and RU486-induced PTL were observed. Several enrichment profiles specific to LPS-induced labor were identified and included pathways involved in bacterial and viral pattern recognition, interferon signaling, death receptor signaling, and acute phase response signaling. A core set of gene changes (95 upregulated, 148 downregulated, Additional file 10) were identified across all models. Process networks analysis on this core set showed enrichment of signaling pathways involved in tissue remodeling (*P* = 1.12 × 10^−3^), muscle contraction (*P* = 1.499 × 10^−2^), cell proliferation (*P* = 1.94 × 10^−7^), apoptosis (*P* = 9.190 × 10^−3^), and NFkB and mitogen-activated protein kinase (MAPK) signaling (*P* = 9.186 × 10^−3^ and 1.747 × 10^−2^ respectively, Fig. 2b). This group included genes that have been shown to be associated with myometrial activation and contraction, including increased levels of prostaglandin-endoperoxide synthase 2 (*Ptgs2*), prostaglandin E synthase 2 (*Ptges2*) [59], and apolipoproteins [60] as well as decreased levels of potassium voltage-gated channels [61], G-protein coupled receptors, and leukemia inhibitory factor receptor, consistent with a compensatory role in the attenuation of myometrial sensitivity to contraction-promoting signals as labor approaches [62–65]. In addition, numerous novel candidates were identified that may play an important, previously unrecognized, role in promoting myometrial contractions, including components of the cytoskeleton machinery, transporters, and alternative potassium channel isoforms. Similar analyses of genes exclusively modulated throughout normal murine gestation (341 upregulated, 260 downregulated, Fig. 2c) showed enrichment of several biological functions associated with pregnancy, such as muscle contraction pathways, including genes encoding for G-protein coupled receptors, guanylate cyclase-activating protein, and dystroglycan; genes involved in gonadotropin regulation : (i.e., family members of the AP-1 family such as *JunB*, *Fos*, and *FosB*), chemotaxis, and inflammation [i.e., chemokine (C-X-C motif) ligand (*Cxcl*) *1*, *Cxcl2*, *Cxcl10*, *Cxcl16*, and *TNF-α*] (Additional file 11).

We next examined those genes changing specifically in the RU486-induced PTL model (286 upregulated, 303 downregulated, Fig. 2d). DEGs unique to this model were primarily associated with tissue remodeling (cell adhesion and proteolysis), consistent with the role of the progesterone/PR axis in regulating extracellular matrix integrity [66]. These genes included those encoding for collagen structural proteins (*Col5a2* and *Col6a4*) and cadherins (*Cdh4*, *Cdh13*, *Cdh17*, *Pcdha11*,* Pcdha12*) as well as remodeling enzymes such as metalloproteinases (*Mmp2*,* Mmp20*,* Mmp27*) and several genes encoding for Adam (a disintegrin and metallopeptidase domain) family members (*Adam5*, *Adam10*, and *Adam19*; Additional file 12).

Analysis of DEGs in the LPS model showed that approximately 80 % (1848/2312) of gene changes were unique to this model and included genes belonging mostly to inflammatory pathways such as interferon signaling (i.e., *Irf7*, *Stat1*, *Stat2*, and *Stat5*, *iNOS*, and *Caspase-1*), Notch signaling (i.e., platelet-derived growth factor B [*Pdgfb*] and *Pdgfr*), platelet endothelium–leucocyte interactions (i.e., *P-selectin*, *E-selectin*, *L-selectin*, and *CD47*), apoptosis mediated by NFkB (including the genes *C-iap1*, *Tnfrsf17*, *Il-15ra*, and *I-κb*), and immune response T_H_17-derived cytokines pathways (Fig. 2e; Additional file 13).

### Progesterone withdrawal induces two waves of myometrial gene expression changes preceding spontaneous term labor

**Fig. 3:**
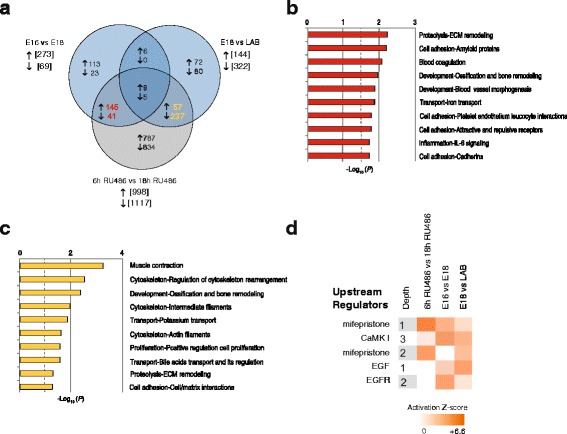
RU486-induced progesterone withdrawal anticipates gene changes observed prior and during labor onset in term gestation. **a** Venn diagram representing overlap between gene changes characterizing term gestation (E16 vs E18 and E18 vs LAB) and genes changing between 6 h and 18 h following RU486 treatment. Two groups of genes were identified, one including 55 % of the genes (187/342) changing between E16 and E18 and 18 h post-RU486 treatment (highlighted in *red*) and a second group including 63 % of the genes (295/466) similarly changing between E18 and LAB and 18 h post-RU486 (highlighted in *yellow*). **b** Process Network Analysis performed on differentially expressed genes similarly changing between E16 and E18 and between 6 h RU486 and 18 h RU486; and (**c**) between E18 and LAB and 6 h RU486 and 18 h RU486. Bar length indicates significance and is equal to the negative logarithm of enrichment *P* value. A *P* value cut-off less than 0.05 (−log_10_ *P* > 1.3) was applied and is indicated by the *dashed line*. **d** Top five putative upstream regulators of differentially expressed genes identified by Causal Network analysis (IPA). Network depth is reported in the first column and indicates the nature of the connection between the expressed genes and the predicted regulator (1 = direct connection, 2 = one intervening regulator, 3 = two intervening regulators). Upstream regulators with a significant overlap (*P* ≤ 0.05) and an activation Z-score ≥ |2.0| were considered significantly activated/inhibited. *LAB* active term labor

Since mice laboring at term or preterm following RU486 treatment exhibited the largest number of shared DEGs, we next sought to investigate the extent and stage at which progesterone signaling was exercising its greatest effect on gene expression. Genes differentially expressed between E16 and E18 and between E18 and LAB were compared with those changing in response to RU486 from 6 h to 18 h, which represented the time frame encompassing the majority of gene changes in this model. Two sets of genes were identified, one including 55 % of the genes (187/342) changing between E16 and E18 and 18 h post-RU486 treatment (early changing genes), and a second group including 63 % of the genes (295/466) similarly changing between E18 and labor and 18 h post-RU486 treatment (late changing genes) (Fig. 3a, FDR < 0.01). Process networks analysis of early progesterone withdrawal response genes showed that these genes are primarily involved in extracellular matrix remodeling (*P* = 6.16 × 10^−3^), cell adhesion (*P* = 6.61 × 10^−3^), and blood vessel morphogenesis (*P* = 1.37 × 10^−2^) (Fig. 3b). Late response genes are involved in muscle contraction (*P* = 3.10 × 10^−4^), regulation of cytoskeleton rearrangement (*P* = 1.96 × 10^−3^), and potassium transport (*P* = 9.87 × 10^−3^) (Fig. 3c). These data provide evidence that progesterone withdrawal mediates two waves of gene expression in the uterus, involved firstly in tissue remodeling, presumably for preparation of the uterus for labor, and subsequently for activation of the uterine contractile machinery. Causal Network Analysis by IPA was then used to identify potential upstream regulators of DEGs detected in each pairwise comparison (Fig. 3d). As expected, the main upstream regulator predicted to modulate gene changes associated with RU486-induced progesterone withdrawal was mifepristone (RU486, Z score_6h RU486 vs 18h RU486_ = 6.6, IPA Causal Network Analysis, Fig. 3d and Additional file 14). RU486 was also predicted to be a major upstream regulator of DEGs between E16 and E18 (Z score_E16 vs E18_ = 2.9) and DEGs between E18 and LAB (Z score_E18 vs LAB_ = 2.2), albeit to a lesser extent. Other top predicted regulators of transcriptional networks in the spontaneous term labor model included calcium/calmodulin-dependent protein kinase I (CaMKI) and epidermal growth factor (EGF) and its receptor (EGFR).

### Identification of time-associated gene expression variations associated with contractile phenotype acquisition during preterm and term labor

**Fig. 4:**
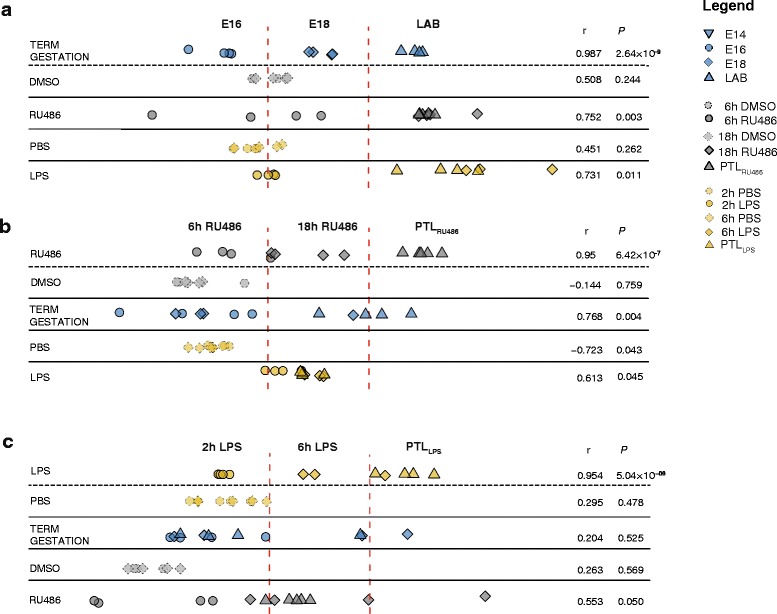
Orthogonal signal correction-partial least squares (OSC-PLS) analysis illustrates gene expression changes associated with progression of term gestation and preterm labor (*PTL*) models. This OSC-PLS analysis describes correlations between time-associated gene changes characterizing term gestation (*blue*), RU486 (*gray*), and lipopolysaccharide (*LPS*) models (*yellow*). **a** Term gestation was used as training set and a positive correlation was found when using either RU486 or LPS models as test sets. **b** Using the RU486 model as a training set, a positive correlation was found with both term and LPS models. **c** When the LPS model was used as the training set, no significant correlation was found with either RU486 or term labor models. Correlation coefficients (*r*) were calculated using Pearson linear correlation (*P* < 0.05). *DMSO* dimethyl sulfoxide, *LAB* active term labor

OSC-PLS [55] analysis was performed to identify time-associated variations in gene expression specific to each parturition model. First, an OSC-PLS model was independently trained on each of the three different murine models: term gestation, PTL induced by RU486, and PTL induced by LPS. The remaining two models were then used as test sets to assess time-associated gene correlations between models. A Pearson’s correlation coefficient (*r*) and its relative *P* value were calculated for each analysis between the gestation time and the predicted scores of the test sets to indicate positive or no correlation between the training and the test set. First, the RU486 and LPS models were used as test sets against genes changing as a function of time in mice spontaneously laboring at term (Fig. 4a). A strong positive correlation was found with the training in both cases (*r* = 0.752, *P* = 0.003 and *r* = 0.731, *P* = 0.011, respectively) indicating consistency between major gene changes associated with progression of gestation and those changes observed with premature parturition induced by RU486 and LPS. Similar results were observed when the RU486 model was used as the training set (Fig. 4b) with a strong positive correlation found between the normal gestation model (*r* = 0.768, *P* = 0.004) and to a lesser extent the LPS model (*r* = 0.613, *P* = 0.045). In contrast, when the LPS model was used as the training set (Fig. 4c) no significant correlation was found with either RU486 (*r* = 0.553, *P* = 0.05) or normal gestational (*r* = 0.204, *P* = 0.525) models, providing evidence that temporal gene changes observed in LPS-induced PTL are unique to this model.

### Temporal transcriptome changes in murine term and preterm labor correlate with human labor-associated myometrial gene changes

While animal models of parturition and PTL are regularly used to infer functional mechanisms in human pathology, the reliability of such inference on a global transcriptome scale has yet to be examined. We therefore compared the transcriptome data generated from each of the murine models used in our study with available human uterine transcriptome data [40]. This study was chosen because it represents the first and only RNA-Seq study published to date using pregnant human myometrium collected at term (>37 weeks) before and during labor. This dataset provides transcriptome changes characterizing human myometrium collected during Caesarean section in women following spontaneous labor (*n*=5, IL) onset, and from women not in labor (*n*=5, NIL), between 38 and 40 weeks of gestation undergoing elective Caesarean section.

**Fig. 5:**
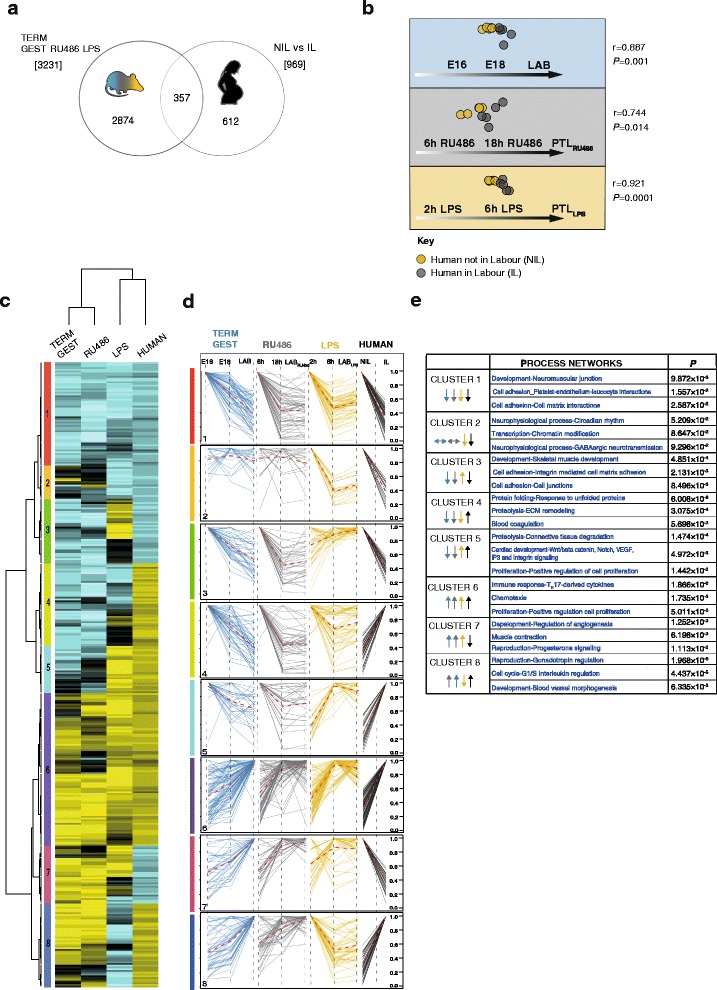
Comparison of murine and human myometrial transcriptome datasets. **a** Venn diagram depicting 357 orthologs significantly changing in the myometrium of murine models of term gestation, RU486-induced, and LPS-induced preterm labor (*PTL*), and at the onset of spontaneous labor in human. The total number of differentially expressed genes identified in murine models and in human samples is reported in square brackets. **b** A total of 357 orthologs were used to perform orthogonal signal correction-partial least squares (OSC-PLS) analysis to test for correlation between murine and human myometrium transcriptome changes associated with activation of contractions and labor. Murine gene expression changes associated with each model of gestation were used as a training set and gene changes characterizing the onset of spontaneous human term labor were tested against each model. *r* Pearson’s correlation coefficient. Correlation between training set and test sets is considered significant if *P* < 0.05. **c** Hierarchical clustering of loadings values from OSC-PLS models allowed the identification of eight clusters (color-coded side bar) of genes with similar expression patterns throughout murine gestation and in human myometrium samples prior to and following the onset of spontaneous term labor. **d** The gene read counts (normalized to the maximum value) belonging to each cluster are displayed separately as gene expression patterns throughout term gestation (*blue*), RU486-induced (*gray*), and lipopolysaccharide (*LPS*)-induced PTL (*yellow*) and during term human labor (*black*). **e** Top networks relative to each cluster identified by Process Network Analysis. Gene patterns associated with each cluster are summarized by the color-coded arrows represented in the first column of the table (*blue*, term gestation; *gray*, RU486 model; *yellow*, LPS model). Enrichment is considered significant if *P* < 0.05

First, edgeR analysis was repeated to identify DEGs with a homogenous FDR threshold of less than 0.001, which was applied to both human and murine datasets. We identified 357 DEGs shared between mouse and human datasets that were selected for further analysis (Fig. 5a, Additional file 15). OSC-PLS modeling was then used to investigate the relationship between time-associated gene changes observed in mouse myometrium and changes associated with the acquisition of a laboring phenotype in human myometrium. Gene expression variations across term murine gestation, and following RU486 or LPS treatment in the mouse, served as training sets against which the corresponding gene changes in human myometrium were tested (Fig. 5b). The OSC-PLS model showed clear separation between human samples collected prior to and following the onset of labor when each of the murine models was alternatively used as the training set. These results indicate the existence of temporal similarities in gene changes characterizing murine and human myometrial evolution to a contractile phenotype regardless of the nature of the gestation (term or preterm). The strongest correlation was observed between human and LPS-induced PTL gene changes. We then examined the relative contribution of each gene to the gestational models by analyzing corresponding loading scores using hierarchical clustering analysis (Fig. 5c, Additional file 16). Human transcriptome changes associated with labor were mostly similar to those gene changes observed in LPS-induced PTL in the mouse, whereas orthologous DEGs in RU486-induced PTL clustered with spontaneous mouse term labor. Temporal dynamics of these gene clusters were then examined in each of the murine models of parturition and human datasets (Fig. 5d). Process Network Analysis (Fig. 5e) of genes downregulated throughout gestation (Cluster 1) showed high representation in cell adhesion pathways. Genes upregulated throughout gestation, belonging to Cluster 6, were strongly enriched in immune response, chemotaxis, and cell proliferation pathways. Two other clusters, 4 and 7, are noteworthy since the direction of labor-associated changes in the human is opposite to all three murine models.

## Discussion

We provide a detailed description of transcriptome changes characterizing the transition of the uterus from a quiescent to a contractile phenotype in three different models of murine parturition: term gestation, PTL induced by functional progesterone withdrawal (RU486), and PTL induced by inflammation (LPS). Further, we compared changes in the mouse myometrial transcriptome with those observed in the human myometrium with labor onset. Our results reveal the relative contribution of hormonal and inflammatory signals to labor onset, and provide further insights into the biology of parturition.

The murine models examined in our study are widely used by the research community to mimic pathophysiological processes of human parturition [32, 67]. Progesterone withdrawal, be it either functional as in the human, or systemic, as in most other mammals, results in the release of the progesterone block and activation of uterine contractions [45, 68]. Infection and/or inflammation are frequently associated with human PTL, accounting for 30 % of all cases [2, 31]. Therefore, bacteria-derived molecules such as LPS are often used to mimic infection/inflammation-induced PTL in animal models [12, 32], which occurs without changes in the levels of circulating progesterone [10, 69]. While the majority of very early PTL cases in humans are associated with infection/inflammation, most late PTL cases are not [3]. Here we show that normal murine labor at term and RU486-induced PTL exhibited similar global gene expression changes, while PTL induced by LPS exhibited a distinctive transcriptome profile. These results are consistent with previous studies indicating differences between the underlying mechanisms of inflammation- and progesterone withdrawal-induced PTL and spontaneous term labor [9, 10, 36].

By identifying genes differentially expressed throughout gestation, our study revealed that the great majority of gene changes are observed before the onset of labor in the mouse. This highlights the importance of temporal transcriptome profiling approaches. Previous human or animal studies comparing samples collected prior to and following labor provide very limited insight into transcriptome changes required specifically for labor onset. Many gene expression changes observed in samples collected during labor likely occur as a consequence of labor or may be due to activation of uterine involution and be erroneously assumed as necessary for the onset of labor [70]. To control for this in our study, samples obtained from each animal model at labor onset were collected immediately following the birth of the first pup.

Cross-comparison of DEGs identified in each model of parturition allowed identification of a “core” gene set likely to be representative of transcriptome changes required for both term and PTL. These genes are overrepresented in pathways associated with cell proliferation, adhesion and tissue remodeling, ion transport, muscle contraction, and NFkB and MAPK signaling. Transcriptome changes unique to the onset of normal term labor were predominately related to muscle contraction, chemotaxis, and inflammation, indicating that the process of normal labor involves an inflammatory component. These data are consistent with the concept that spontaneous labor in the mouse is initiated by fetal signals that induce progesterone withdrawal via uterine NFkB activation and subsequent inflammatory pathway activation [5, 6]. However, although spontaneous labor is associated with a chemokine-driven influx of inflammatory cells into the myometrium, depletion of various immune cells subsets does not block labor [24–26, 28]. Moreover, activation of pro-inflammatory pathways is sufficient but not required for cervical ripening [28, 71, 72]. These data allude to the possibility that inflammatory pathway activation in the mouse uterus at the time of labor is a side effect of the mechanism leading to progesterone withdrawal. Further evidence for this was generated when we examined those genes changing specifically in the RU486-induced PTL model. Our analyses show that term gestation and RU486-induced PTL models share around 60 % of similarly regulated genes, of which the majority are prematurely modulated by RU486 treatment prior to labor onset in this model. DEGs unique to this model were, however, primarily associated with tissue remodeling (cell adhesion and proteolysis), consistent with the role of progesterone/PR axis in regulating extracellular matrix integrity [66]. The absence of inflammatory gene network upregulation in the uterus following RU486-induced labor, taken together with previous studies showing that knockout of PR in myocytes does not affect inflammatory gene expression [66] and that RU486-induced labor does not involve infiltration of macrophages or neutrophils [70], provides further evidence that the acquisition of a contractile phenotype can be attained in the absence of inflammatory gene activation or synthesis of pro-inflammatory cytokines.

While activation of inflammation via progesterone withdrawal does not appear to be a requisite for acquisition of a contractile phenotype in the mouse, much evidence suggests that inflammation can still promote a laboring phenotype in pathological situations, particularly infection/inflammation-induced PTL. For example, bacteria and their products can robustly induce preterm birth in the absence of progesterone withdrawal [10, 69]. We have also previously shown that myometrial inflammatory and contractile gene activation via AP-1 is sufficient to cause PTL in the mouse at gestation day 16, when circulating progesterone levels are high [11]. In vitro studies have also shown that LPS may directly promote myocyte contraction via activation of the Rho/ROCK signaling pathways [73]. In this study we identified a set of genes uniquely upregulated with increasing proximity to labor following LPS treatment that were overrepresented in multiple inflammatory pathways. While these changes may represent unique mechanisms of contractile activation via inflammatory gene modulation, they may alternatively represent unrelated acute inflammatory response to LPS treatment [36] and still harbor the potential to cause maternal and, importantly, fetal damage [9, 74–78].

We identified two sets of genes shared between normal and RU486 models, whose expression changed either early (E16 vs E18) or late (E18 vs LAB) in gestation. Process Network Analysis showed that the earlier responding genes were primarily involved in tissue remodeling, cell adhesion, and blood vessel morphogenesis, whereas the later responding genes were involved in muscle contraction, regulation of cytoskeleton rearrangement, and potassium transport. These data provide evidence that progesterone withdrawal mediates two waves of gene expression in the uterus involved firstly in tissue remodeling, presumably for preparation of the uterus for labor, and subsequently for activation of the uterine contractile machinery.

Considering E16 as the start point of the time-course experiment, gene expression changes associated with ongoing gestation were acquired across a different time scale in each mouse model. OSC-PLS modeling of temporal gene changes observed throughout murine gestation allowed the investigation of similarities in the transcriptome profiles underlying labor onset. This analysis showed that gene changes acquired throughout a term gestation also occur in a similar fashion in the RU486- and LPS-induced PTL models, although within a shorter time frame. Second, by using each murine model alternatively as a training set, it was possible to test whether similar or different genes were important for the acquisition of a laboring phenotype in each model. These results showed that similar transcriptome changes govern the onset of both term and RU486-induced PTL. In contrast, time-dependent transcriptome changes induced by LPS treatment did not correlate with term gestation or RU486 models, suggesting the existence of a specific set of genes controlling the onset of labor in the LPS model.

To examine how our murine models of parturition relate to the human, we re-analyzed an available RNA-Seq dataset reporting transcriptome changes characterizing human myometrium prior to and following spontaneous term labor [40]. Analysis of correlations between mouse and human transcriptome changes associated with labor showed high similarity in gene expression patterns in both species, further supporting the validity of the murine model in the investigation of transcriptional mechanisms of parturition. OSC-PLS modeling allowed assessment of the relative contribution of inflammatory (LPS) or progesterone-regulated (RU486) pathways to human labor. Although a significantly positive correlation was found between human and all murine models, human myometrial transcriptome changes at labor most closely resembled the mouse LPS model. Examination of the temporal gene changes associated with murine gestation and labor and with gene changes captured at labor onset in human allowed the identification of two clusters (Cluster 1 and 6) that comprised genes with similar expression profiles in both species. Genes belonging to Cluster 1, downregulated throughout gestation, were highly represented in cell adhesion pathways likely to be progesterone-dependent [66]. Cluster 6 included genes consistently upregulated in both mouse and human myometrium, most of which were inflammatory genes. This is consistent with previous reports describing activation of inflammation prior to labor onset in the human [79] and previous in vitro studies performed by our group [66] suggesting that inflammation may represent a mechanism to antagonize progesterone-induced gene expression. We identified two other clusters, 4 and 7, that comprised genes whose direction of change within the gestational time course in the human was opposite to all three murine models. We speculate this may be because the human samples were collected well after the onset of labor, and thus may reflect gene expression changes induced by labor itself or by the initiation of involution mechanisms necessary for remodeling the uterus post-delivery [40]. Additionally, we showed that those genes similarly modulated in the LPS-induced murine PTL model and in human labor (Cluster 5) were enriched in proteolysis and cell proliferation pathways. These data indicate that a significant percentage of gene expression changes observed in human at labor onset are likely to represent the activation of involution pathways rather than pathways involved in activation of myometrial contractions.

Comparison between murine models of labor with human-derived data highlighted the existence of similarities in temporal gene changes acquired towards labor. Moreover, these data provide further evidence that the mechanism underlying human labor onset involves progesterone signaling that may occur via functional progesterone withdrawal mediated by changes in PR isoform expression [19, 20, 80], but point toward a more prominent role for inflammatory pathway activation in human labor onset [81].

## Conclusions

Our data are concordant with the paradigm that murine labor is initiated by progesterone withdrawal through fetal signaling that involves induction of inflammatory transcription factors and subsequent inflammatory gene activation in the uterus. However, parturition can be induced by progesterone withdrawal alone and inflammation is not required for the acquisition of the laboring phenotype in the mouse. Conversely, LPS activation of inflammation is sufficient to override the repressive effects of high circulating concentrations of progesterone and induce labor in the mouse. Human myometrial transcriptome changes at the time of labor most closely resemble the mouse LPS model, indicating a dominant role for inflammatory pathways in human labor. These results have important implications for the understanding of the underlying mechanism of labor onset and the development of future prevention strategies for preterm birth.

## Abbreviations

ADAM, a disintegrin and metallopeptidase domain; AP-1, activator protein 1; CaMKI, calcium/calmodulin-dependent protein kinase I; Cdh, cadherins; COX-2, cyclooxygenase-2; Cxcl, chemokine (C-X-C motif) ligand; DEGs, differentially expressed genes; DMSO, dimethyl sulfoxide; E, embryonic day; EGF, epidermal growth factor; FDR, false discovery rate; hpi, hours post injection; IL, in labor; IPA, Ingenuity® Pathway Analysis; Irf, interferon; LAB, active term labor; LPS, lipopolysaccharide; MAPK, mitogen-activated protein kinase; Mmp, metalloproteinase; NFkB, nuclear factor kappa light chain enhancer of activated B cells; NIL, not in labor; OSC-PLS, orthogonal signal correction-partial least squares; PBS, phosphate-buffered saline; PCA, principal component analysis; PDGF, platelet-derived growth factor; PGF_2α_, prostaglandin F2 alpha; PR, progesterone receptor; Ptges2, prostaglandin E synthase 2; Ptgs2, prostaglandin-endoperoxide synthase 2; PTL, preterm labor

## Additional files

Additional file 1 List of the total number of reads generated for each sample using RNA-Seq. (XLSX 10 kb) Additional file 2: Supplementary methods used for outlier detection. (DOCX 15 kb) Additional file 3: Complete RNA-Seq dataset as Excel file. (XLSX 34680 kb) Additional file 4: Loadings derived from the OSC-PLS modeling of the 4142 DEGs detected in three models of parturition (term gestation, RU486-induced PTL, and LPS-induced PTL). (XLSX 256 kb) Additional file 5: PCA of transcriptome profiles of murine myometrium collected throughout (a) term gestation, (b) following DMSO/RU486 intraperitoneal injection, and during RU486-induced preterm labor (LAB_RU486_), (c) following PBS/LPS intrauterine injection and during LPS-induced PTL. *n* ≥ 4 biological replicates for each experimental group. (PDF 949 kb) Additional file 6: Venn diagrams depicting number of genes differentially expressed in all pairwise comparisons across term gestation (a), RU486 (b), and LPS models (c). FDR < 0.001. *n* ≥ 4 biological replicates for each experimental group. (PDF 1024 kb) Additional file 7: Gene Ontology Process Network Analysis by Metacore® performed on genes exclusively changing 6 h following LPS treatment and during LPS-induced PTL but not changing throughout term gestation or the RU486-induced PTL model. Bar length indicates significance and is equal to the negative logarithm of enrichment *P* value. A *P* value cut-off less than 0.05 (−log_10_ *P* > 1.3) was applied and is indicated by the dashed line. (PDF 119 kb) Additional file 8: Canonical pathways enrichment calculated by Ingenuity Pathway Analysis. A total of 46 pathways were detected as significantly enriched with genes differentially expressed in the pairwise comparisons indicated in the first row of the table (*P* < 0.05). First column on the left indicates Gene Ontology name associated with the canonical pathway. Log_10_ *P* values are reported for each pathway. (XLS 38 kb) Additional file 9: Canonical Pathway enrichment by IPA®. DEGs as detected in the following pairwise comparisons were analyzed by IPA to identify canonical pathways highly represented within each comparison: 2 h LPS vs 6 h LPS, 2 h LPS vs LAB_LPS_, 6 h RU486 vs 18 h RU486, 6 h RU486 vs LAB_RU486_, 18 h vs LAB_RU486_, E16 vs E18, E16 vs term labor (LAB), and E18 vs LAB. Hierarchical clustering of significantly enriched pathways based on their activation Z-score was used to assess for similarity in enrichment profiles between different models. Activation Z-score > |2|, *P* < 0.05. (PDF 196 kb) Additional file 10: List of core set of genes associated with all models of parturition. (XLS 258 kb) Additional file 11: List of genes exclusively changing throughout normal murine gestation. (XLS 584 kb) Additional file 12: List of genes exclusively changing in the RU486-induced PTL model. (XLS 573 kb) Additional file 13: List of genes exclusively changing in the LPS-induced PTL model. (XLS 1767 kb) Additional file 14: Z-scores derived from Causal Network Analysis by IPA®. (XLS 75 kb) Additional file 15: Ensembl gene annotation for the 357 gene orthologs differentially expressed across murine gestation and during human labor. (XLSX 72 kb) Additional file 16: Loadings values derived from the OSC-PLS modeling of the 357 human–mouse gene orthologs. (XLSX 39 kb)
