# Supplementary material for: Modeling hormonal and inflammatory contributions to preterm and term labor using uterine temporal transcriptomics
Source: BMC Med. 2016 Jun 13;14:86. doi: 10.1186/s12916-016-0632-4 (PMC4904357; doi:10.1186/s12916-016-0632-4)
Supplement: Additional file 2: — Supplementary methods used for outlier detection. (DOCX 15 kb) [file 12916_2016_632_MOESM2_ESM.docx]

**Additional data 2**: Supplementary methods used for outlier detection.

**Outlier detection for multidimensional data**

We developed a new method for the detection of outliers in a multivariate dataset. This method takes as input the data and the relative information about the association of each sample to each group, and it gives as output the probability of each sample to not be an outlier. This probability is calculated comparing the Euclidean distance of each samples (distance *x*) with the Euclidean distance of all other samples belonging to the same group (distances *A=[a_1_,a_2_,a_3_,a_4_,…,a_n_]*) from the centroid of the group. The probability will be then given by the probability that the distance *x* belong to a Gaussian distribution with mean *µ=mean(A)* and variance *σ^2^=(n-1)/n * variance(A)*.

The R script of the function is provided here below followed by an example with the famous iris dataset.

outlier.detection = function(data,class){

class=as.numeric(as.factor(class))

n=max(class)

pro=rep(NA,n)

me=NULL

TOTdist=rep(NA,n)

med=matrix(nrow=n,ncol=ncol(data))

nn=nrow(data)

for(j in 1:nn){

dataCV=data

classCV=class

for(i in 1:n){

da=dataCV[classCV==i,]

med[i,]=colMeans(da)

}

CVdist=NULL

for(i in 1:nrow(data)){

mediana=med[class[i],]

CVdist[i]=dist(rbind(mediana,data[i,]))

}

pro[j]=pnorm(CVdist[j], mean=mean(CVdist[-j]),

sd=sqrt(sd(CVdist[-j])^2*(nn-1)/nn), lower.tail=FALSE)

}

pro

}

# Example ################

data=iris[,-5]

labels= rep(1:3,each=50)

pca=prcomp(data)$x

pp=outlier.detection(data,labels)

plot(pca,col=labels,pch=(pp<0.05)+1)

##########################
