# Supplementary figures and images for: Modeling hormonal and inflammatory contributions to preterm and term labor using uterine temporal transcriptomics
Source: BMC Med. 2016 Jun 13;14:86. doi: 10.1186/s12916-016-0632-4 (PMC4904357; doi:10.1186/s12916-016-0632-4)

a

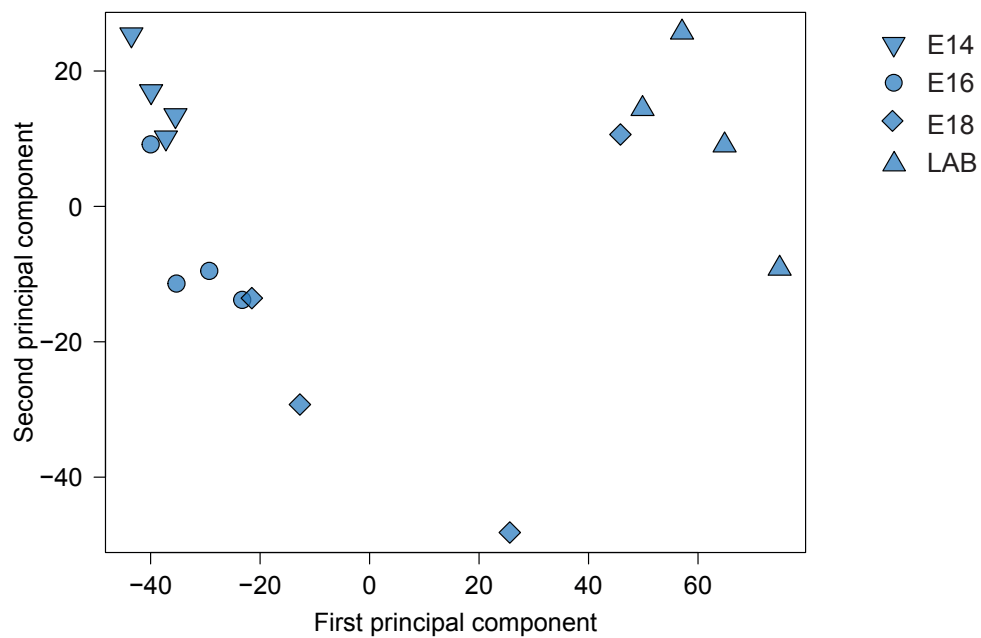

b

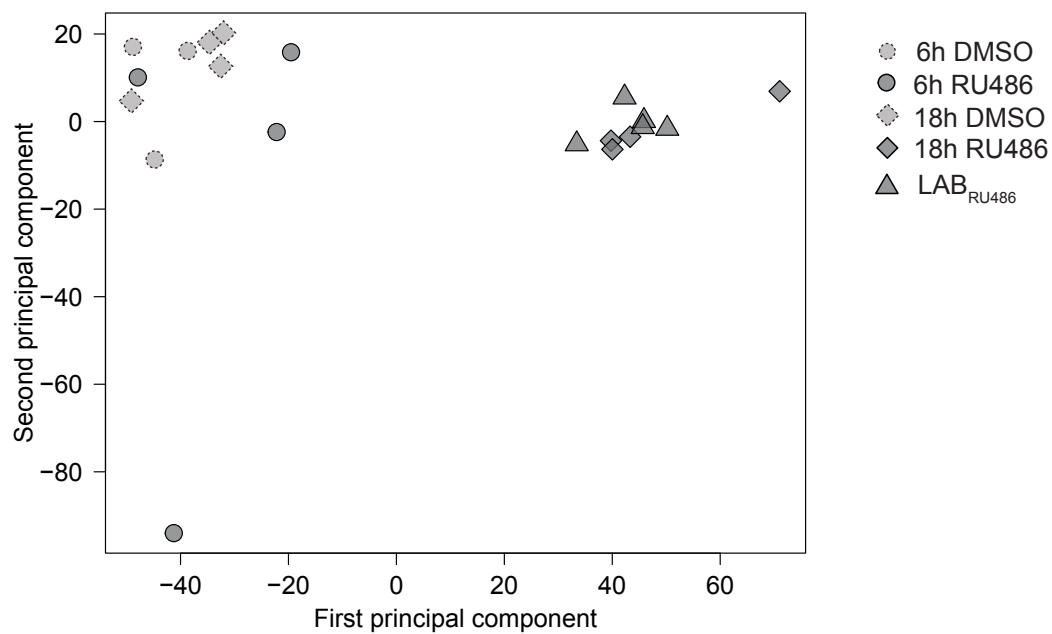

Supplement: Additional file 5: — PCA of transcriptome profiles of murine myometrium collected throughout (a) term gestation, (b) following DMSO/RU486 intraperitoneal injection, and during RU486-induced preterm labor (LABRU486), (c) following PBS/LPS intrauterine injection and during LPS-induced PTL. n ≥ 4 biological replicates for each experimental group. (PDF 949 kb) [file 12916_2016_632_MOESM5_ESM.pdf]

a

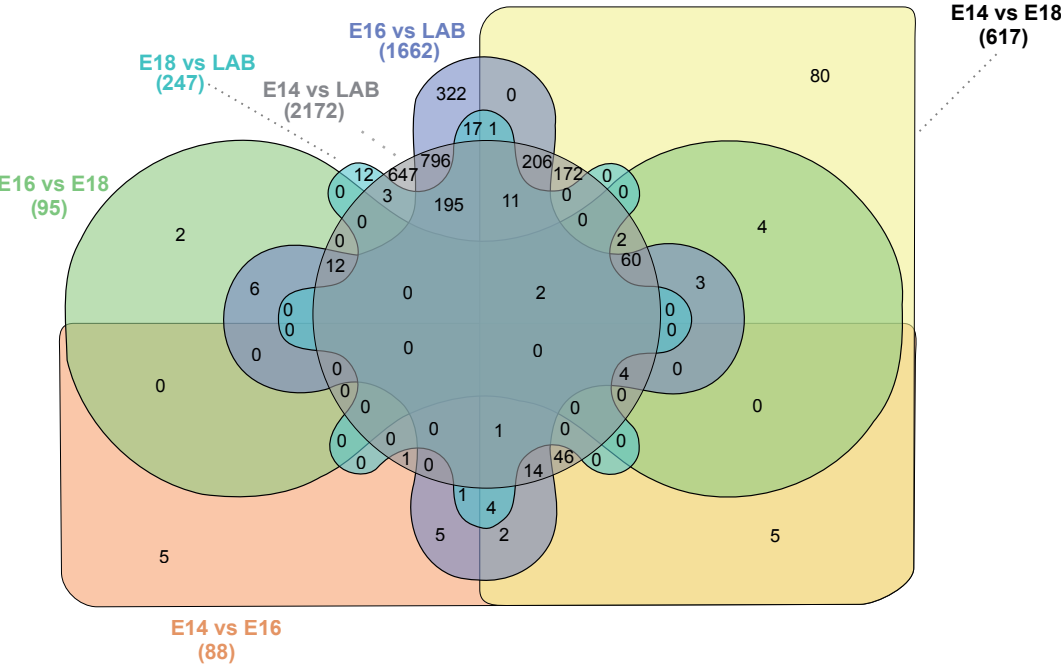

b

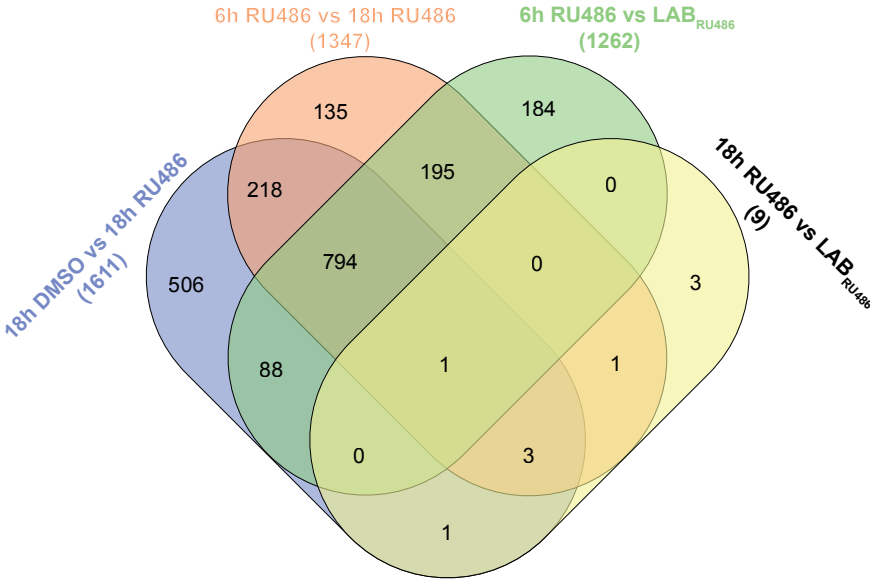

c

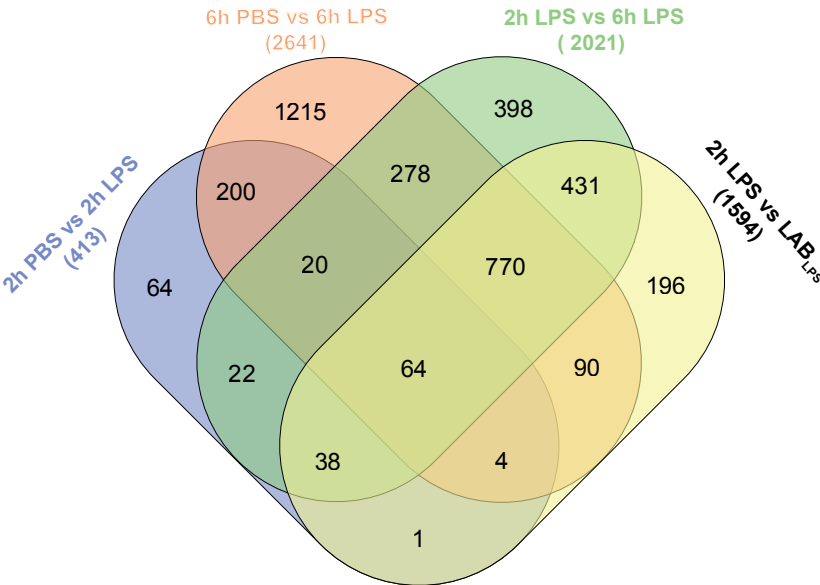

Supplement: Additional file 6: — Venn diagrams depicting number of genes differentially expressed in all pairwise comparisons across term gestation (a), RU486 (b), and LPS models (c). FDR < 0.001. n ≥ 4 biological replicates for each experimental group. (PDF 1024 kb) [file 12916_2016_632_MOESM6_ESM.pdf]

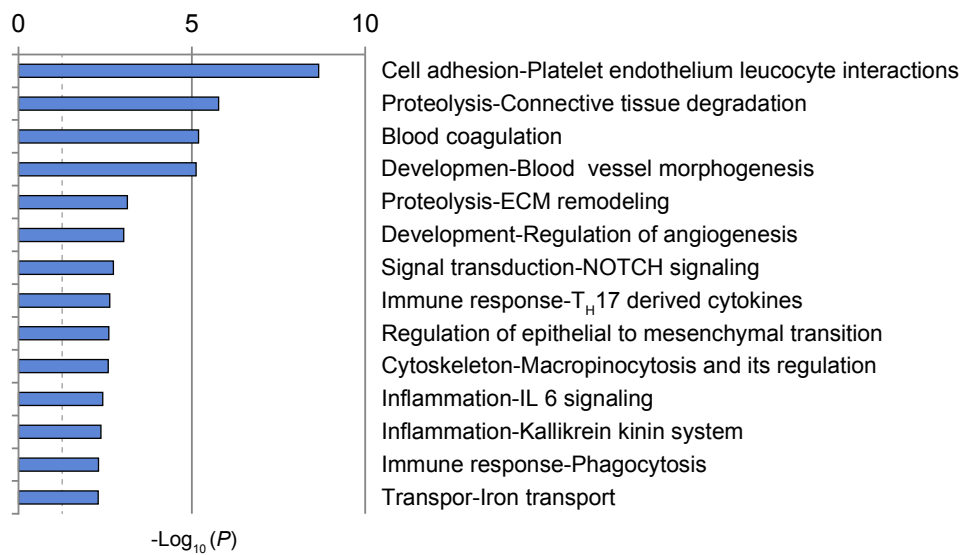

Supplement: Additional file 7: — Gene Ontology Process Network Analysis by Metacore® performed on genes exclusively changing 6 h following LPS treatment and during LPS-induced PTL but not changing throughout term gestation or the RU486-induced PTL model. Bar length indicates significance and is equal to the negative logarithm of enrichment P value. A P value cut-off less than 0.05 (−log10 P > 1.3) was applied and is indicated by the dashed line. (PDF 119 kb) [file 12916_2016_632_MOESM7_ESM.pdf]

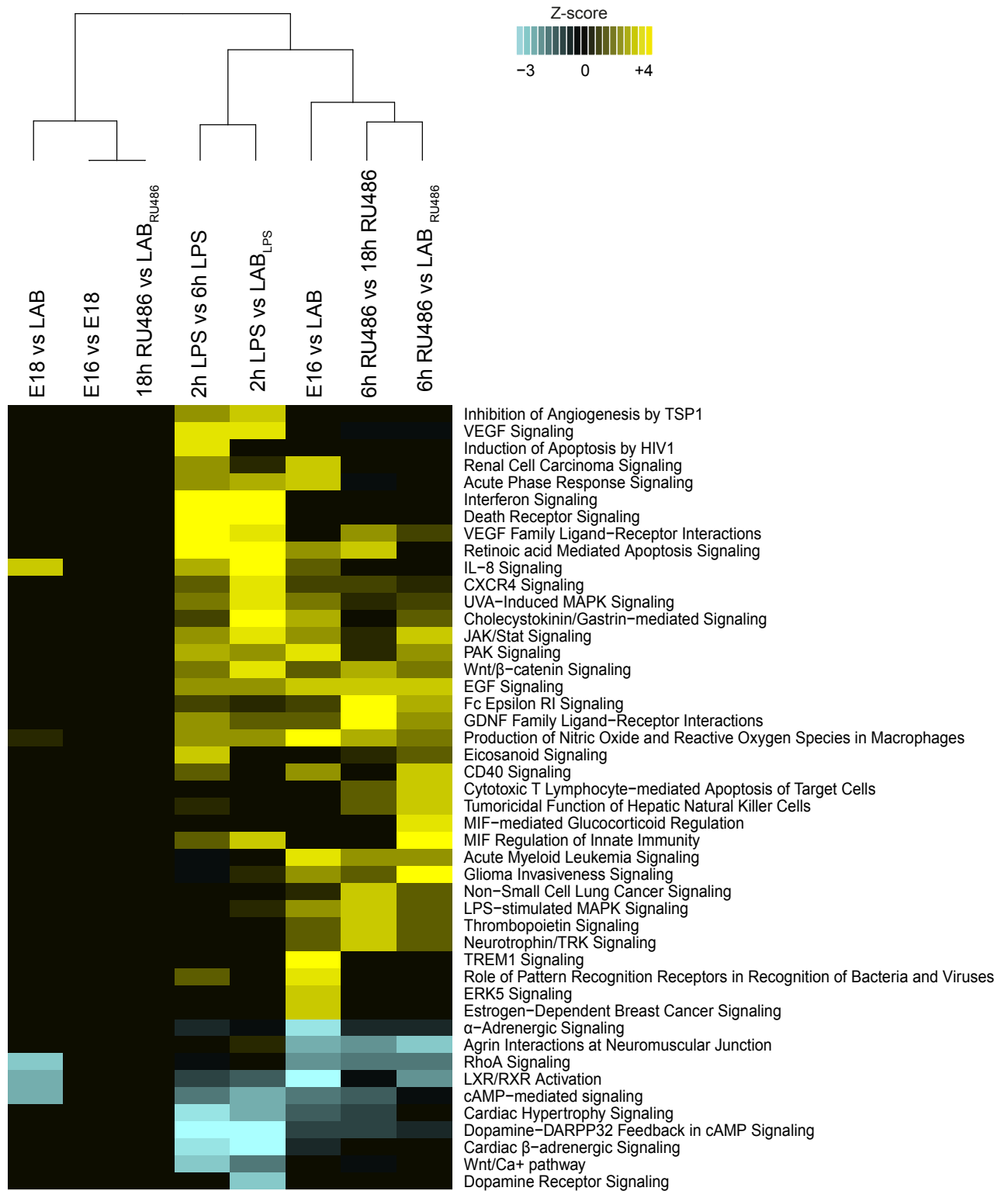

Supplement: Additional file 9: — Canonical Pathway enrichment by IPA®. DEGs as detected in the following pairwise comparisons were analyzed by IPA to identify canonical pathways highly represented within each comparison: 2 h LPS vs 6 h LPS, 2 h LPS vs LABLPS, 6 h RU486 vs 18 h RU486, 6 h RU486 vs LABRU486, 18 h vs LABRU486, E16 vs E18, E16 vs term labor (LAB), and E18 vs LAB. Hierarchical clustering of significantly enriched pathways based on their activation Z-score was used to assess for similarity in enrichment profiles between different models. Activation Z-score > |2|, P < 0.05. (PDF 196 kb) [file 12916_2016_632_MOESM9_ESM.pdf]
